# A Mobile App–Based Intervention Program for Nonprofessional Caregivers to Promote Positive Mental Health: Randomized Controlled Trial

**DOI:** 10.2196/21708

**Published:** 2021-01-22

**Authors:** Carme Ferré-Grau, Laia Raigal-Aran, Jael Lorca-Cabrera, Teresa Lluch-Canut, Maria Ferré-Bergadà, Mar Lleixá-Fortuño, Montserrat Puig-Llobet, Maria Dolores Miguel-Ruiz, Núria Albacar-Riobóo

**Affiliations:** 1 Department of Nursing Universitat Rovira i Virgili Tarragona Spain; 2 Department of Nursing Universitat Rovira i Virgili Tortosa Spain; 3 Department of Public Health, Mental Health and Maternal-Child Nursing, School of Nursing University of Barcelona Barcelona Spain; 4 Department of Computer Engineering and Mathematics Universitat Rovira i Virgili Tarragona Spain; 5 Territorial Health Services of Terres de l’Ebre Catalan Health Institute Tortosa Spain; 6 Department of Mental Health, Campus Docent Sant Joan de Déu-–Fundació Privada University of Barcelona Barcelona Spain

**Keywords:** clinical trial, caregiver, mobile phone app, intervention program, positive mental health, mobile health, health promotion, randomized controlled trial, nursing, caregiving, technology

## Abstract

**Background:**

While nonprofessional caregivers often experience a sense of fulfillment when they provide care, there is also a significant risk of emotional and physical burnout. Consequently, this can negatively affect both the caregiver and the person being cared for. Intervention programs can help empower nonprofessional caregivers of people with chronic diseases and develop solutions to decrease the physical and psychological consequences resulting from caregiving. However, most clinically tested intervention programs for nonprofessional caregivers require face-to-face training, and many caregivers encounter obstacles that hinder their participation in such programs. Consequently, it is necessary to design internet-based intervention programs for nonprofessional caregivers that address their needs and test the efficacy of the programs.

**Objective:**

The aim of this study was to evaluate the effectiveness of a smartphone app–based intervention program to increase positive mental health for nonprofessional caregivers.

**Methods:**

This study was a randomized controlled trial of 3 months’ duration. A total of 152 caregivers over 18 years of age with a minimum of 4 months’ experience as nonprofessional caregivers were recruited from primary health care institutions. Nonprofessional caregivers were randomized into two groups. In the intervention group, each caregiver installed a smartphone app and used it for 28 days. This app offered them daily activities that were based on 10 recommendations to promote positive mental health. The level of positive mental health, measured using the Positive Mental Health Questionnaire (PMHQ), and caregiver burden, measured using the 7-item short-form version of the Zarit Caregiver Burden Interview (ZBI-7), were the primary outcomes. Users’ satisfaction was also measured.

**Results:**

In all, 113 caregivers completed the study. After the first month of the intervention, only one factor of the PMHQ, F1–Personal satisfaction, showed a significant difference between the groups, but it was not clinically relevant (0.96; *P*=.03). However, the intervention group obtained a higher mean change for the overall PMHQ score (mean change between groups: 1.40; *P*=.24). The results after the third month of the intervention showed an increment of PMHQ scores. The mean difference of change in the PMHQ score showed a significant difference between the groups (11.43; *P*<.001; *d*=0.82). Significant changes were reported in 5 of the 6 factors, especially F5–Problem solving and self-actualization (5.69; *P*<.001; *d*=0.71), F2–Prosocial attitude (2.47; *P*<.001; *d*=1.18), and F3–Self-control (0.76; *P*=.03; *d*=0.50). The results of the ZBI-7 showed a decrease in caregiver burden in the intervention group, although the results were inconclusive. Approximately 93.9% (46/49) of the app users indicated that they would recommend the app to other caregivers and 56.3% (27/49) agreed that an extension of the program’s duration would be beneficial.

**Conclusions:**

The app-based intervention program analyzed in this study was effective in promoting positive mental health and decreasing the burden of caregivers and achieved a high range of user satisfaction. This study provides evidence that mobile phone app–based intervention programs may be useful tools for increasing nonprofessional caregivers’ well-being. The assessment of the effectiveness of intervention programs through clinical trials should be a focus to promote internet-based programs in health policies.

**Trial Registration:**

ISRCTN Registry ISRCTN14818443; http://www.isrctn.com/ISRCTN14818443

**International Registered Report Identifier (IRRID):**

RR2-10.1186/s12889-019-7264-5

## Introduction

The increase in life expectancy has contributed to the progressive aging of the population, which has modified the epidemiological pattern, marked by an increase in chronic diseases. Chronic noncommunicable diseases are the main cause of mortality and morbidity in the world, accounting for the death of 38 million people worldwide each year [1]. In Spain, chronic diseases constitute one of the main public health problems, as they are the cause of the increase in morbidity, disability, and impairment, with a resulting impact on the lives of both patients and their caregivers [2].

In Spain, nonprofessional caregivers are on the front line of care for people with physical and/or psychological dependencies. The most common type of nonprofessional caregiver is a family member, althought there is an increase in the number of caregivers contracted by families or the person being cared for who are not trained health care professionals [3,4]. According to the national and international literature, there is a consensus that people who take on the role of nonprofessional caregivers—continuously and/or over a long period of time—must carry out multiple and/or complex tasks, which often leads to feelings of discouragement and stress [5,6]. Evidence suggests that nonprofessional caregivers may experience negative symptoms such as sleep disturbance, fatigue, depression, and anxiety [7,8]. Experiencing such difficulties involves a process that continuously tests their physical capacity and positive mental health, which often leads to overburdening and/or caregiver burnout [8-10].

Since the caregiver’s role is an important element in ensuring the well-being of the person being cared for [11], intervention in this population group becomes a priority. There are intervention and/or support programs that have been positively evaluated [12-15], although most of them are based on in-person training. However, research has shown that many caregivers underutilize the supports available to them and instead try to handle everything by themselves [16,17]. The barriers that hinder access to in-person support programs include a lack of coordinated home care services [18], the multiple occupations and intensive dedication required by family care [19], geographical or transportation limitations, and the caregivers’ own health problems [20,21]. These obstacles often make access to these resources difficult. The development and evaluation of mobile health (mHealth) tools, supervised by health professionals and adapted to the time constraints of caregivers, might be a useful strategy for an intervention program aimed at this population group.

We live in a digital age, in which digital literacy is a necessary competence for the practice of any profession and even more so in the field of health care. Nurses must develop new digital strategies to carry out their professional activities [22]. Traditional models of health care are changing with the development of information and communication technologies (ICTs) and the incorporation of mHealth solutions. Digital health intervention programs have had a significant impact on the care of chronic diseases, providing access to electronic health records, apps, and health portals [21,23-26].

Apps for smartphones and tablets have become indispensible and complementary tools to health care [27,28]. They provide an opportunity to lead ICT-based projects to empower people, teach them how to manage their health, improve their quality of life, and achieve well-being [21,22]. A recent review highlighted the importance of implementing new technologies in health care policies—specifically, in that case study, telemedicine [29]. It stressed that further studies should be carried out aimed at a consistent analysis and follow-up of patients after such intervention programs as a strategy to demonstrate that they are necessary [30].

There are currently more than 325,000 health apps available for health system users to download and interact with on their mobile devices. These tools can help improve the possibilities for continuity of care for the population, optimize existing resources, and increase the quality of care [28,31].

A systematic review by Lorca-Cabrera et al [21] concluded that there are very few studies on the evaluation of apps aimed at improving the health and welfare of caregivers. This reality, aggravated by the lack of regulation of effective apps for this purpose, can lead to health issues for patients and their caregivers due to potentially erroneous or inaccurate information provided by these apps [32,33]. It is therefore necessary to design and evaluate the apps in which intervention program for caregivers are conducted and measure their effectiveness. In this way, caregivers will benefit from the full potential of the apps to manage their self-care and improve their quality of life [34].

This study was based on a previous project in which a website [25,35] was developed for caregivers of patients with chronic diseases [25]. Given the evidence mentioned above, this study aimed to evaluate a digital intervention program of care for caregivers, using a mobile app that promotes positive mental health and/or reduces overburdening. The theoretical basis for the promotion of caregivers’ health was Lluch-Canut’s positive mental health assessment model, along with her decalogue of practical recommendations [36,37]. The protocol of this clinical trial study was previously published [38].

## Methods

### Aims

This study aimed to evaluate the effectiveness of a smartphone app–based intervention program compared with a standard intervention program for caregivers in primary health care institutions. The usability and satisfaction of the app were a focus of this study as well. The hypothesis was that an app-based intervention program would improve caregivers’ mental health and decrease their burden.

### Design

**Figure 1:**
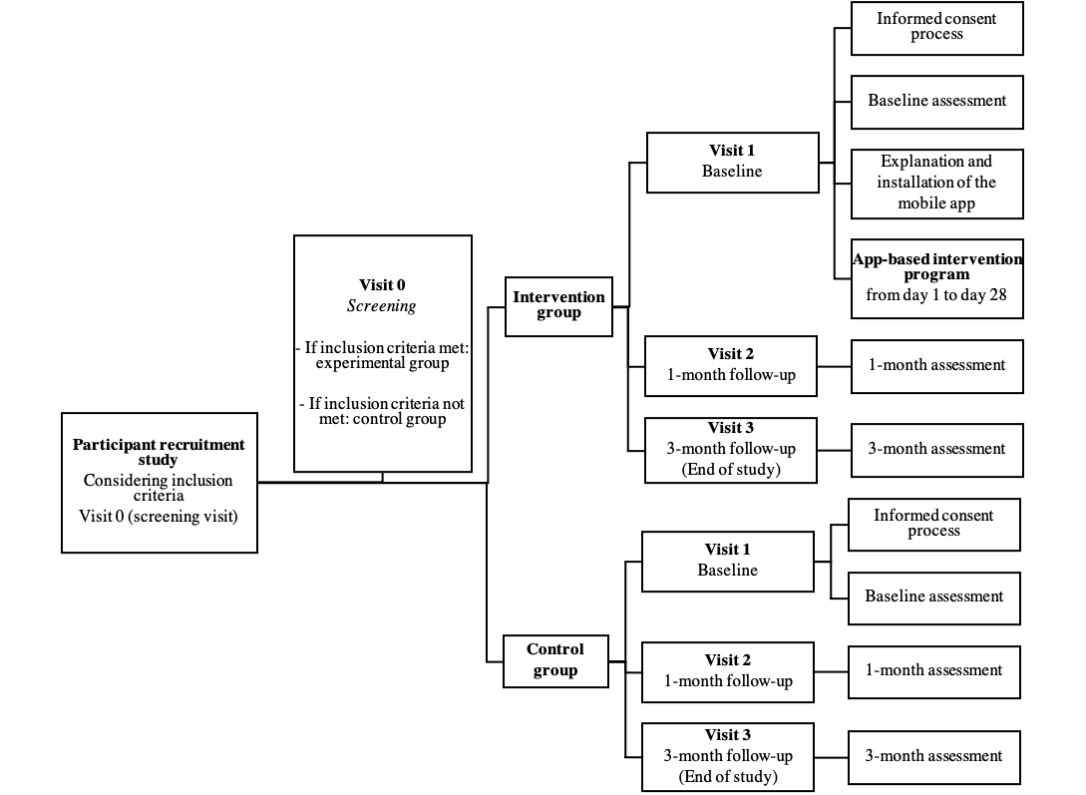
Participant flowchart.

A randomized controlled trial (RCT) was conducted. Participants were randomly assigned to either the experimental group or the control group (Figure 1). The RCT was registered in the International Standard Randomized Controlled Trial Number registry (ISRCTN: 14818443; May 24, 2019).

### Sample Size Calculation

The study aimed to show differences between an app-based intervention and standard intervention with a standardized effect size (Cohen *d*) of 0.33 or larger. A standardized effect of 0.33 can be considered the lowest limit of a moderate clinical effect [39] and is based on a meta-analysis of well-being intervention research [40] and a recent RCT [41]. The total population of caregivers was uncertain, so the sample size was calculated considering an α risk of .05 and a β risk of .20. The minimum number of participants required to obtain evidential data of the results was 108 subjects, with 54 subjects each in the experimental and control groups. An estimated 30% loss to follow-up was taken into account for each group and a difference greater than or equal to 10 was recognized as statistically significant.

### Recruitment

Primary health care is the first point of access to the public health system in Catalonia, Spain. The rest of the services in the system can be accessed by referral from primary care, except for medical emergencies, which can be accessed directly in the event of urgent need. The primary care center is the place for on-site care where people have to go when they have a health problem or want to prevent an illness. It provides diagnosis and care for the main health problem; health and social care; health promotion services; preventive, curative, and rehabilitative care; home care service; urgent or continuous care; and sexual health care. Primary care services are part of the basic common core portfolio, which are services fully covered by public funding.

The eligible caregivers were recruited by nurses from 7 primary health care centers in central and southern Catalonia. Each nurse invited caregivers they knew who met the criteria to participate in the study. The inclusion criteria for the participants were as follows: (1) primary or secondary nonprofessional caregiver of someone with a chronic disease; (2) over 18 years of age; (3) minimum of 4 months of experience as a caregiver; (4) knowledge of Spanish or Catalan (the app was available in both languages and the user could choose his/her preference); (5) user of a mobile device and the WhatsApp mobile app; (6) access to a mobile device with an Android operating system and internet access; and (7) signature of informed consent.

### Randomization and Blinding

The online randomization procedure was carried out on an individual basis. Caregivers who agreed to take part in the study—who had a phone that supported the app and met the inclusion criteria—were randomly assigned. The randomization was stratified by the primary health center attended (7 centers in total), gender, age, and perceived level of well-being. A computer program allocated the participants using a generated randomization list. Given the nature of the study, it was not possible to blind the caregivers and the professionals.

### Details of the Intervention and Control Groups

Both study groups received the same standard intervention for caregivers by the nursing staff in the primary health care centers. Every year, the nurses assess the burden of the caregivers using the validated Zarit Burden Interview tool. If they identify a high level of burden, they refer the caregiver for psychological intervention. There is no specific protocol or intervention to promote positive attitudes toward caregiving.

#### Control Group

Participants in the control group received the standard intervention for caregivers by the nurses at their primary health care center of reference.

#### Intervention Group

Caregivers in the intervention group received the standard nursing care in addition to a free smartphone app (the TIVA app), which involved a 28-day intervention program. The researcher had to register the app and set a starting date for the intervention program, as agreed upon by the caregiver and the nurse in charge. During this period of time, the caregiver had the smartphone app active on his or her mobile device.

**Figure 2:**
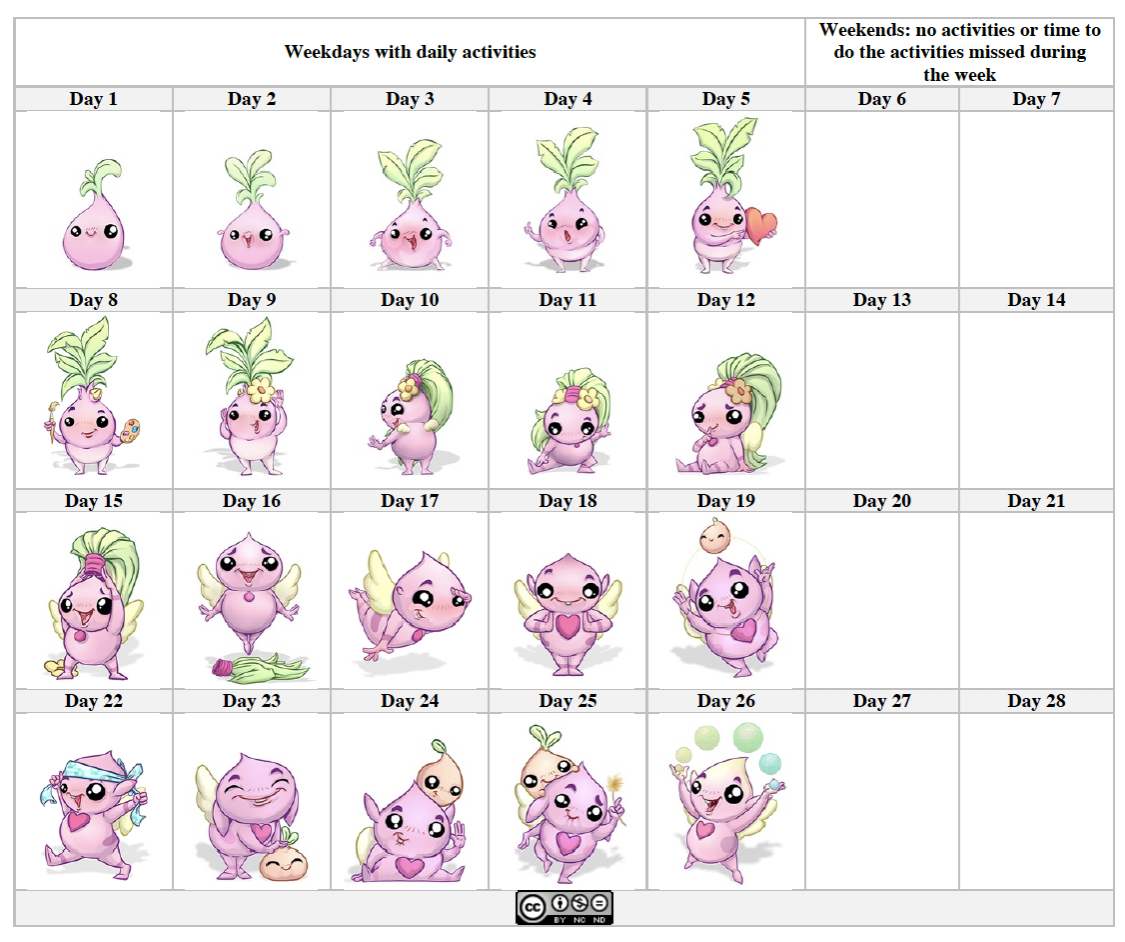
Evolution of the main character of the TIVA app.

In the intervention program, the TIVA app offered participants an activity daily from Monday to Friday. These activities were related to the decalogue of positive mental health described by Lluch-Canut [36]. The decalogue includes 10 recommendations to promote positive mental health. For each recommendation, 2 activities were created by a group of experts who were part of the research group. After carrying out each activity, the caregiver expressed whether it was useful or not. The app provided a motivational quote every day and asked the caregiver, “Hello, how are you today?” Although there were no activities during the weekend, the app still recommended that caregivers visit the website developed in a previous related project [35]. The final activity offered caregivers an opportunity to register on the app’s website. This allowed them to be connected to other caregivers and to have access to any news posted there. The app includes gamification entailing an ad hoc character named TIVA—from the Spanish word “posiTIVA,” meaning “positive.” This character grows up and changes every time the caregiver performs the daily activity (Figure 2).

### Data Collection

The study was performed from September 2019 to November 2019. For the intervention group, primary outcome data were collected through the app, and secondary outcome data were collected by nurses through ad hoc questionnaires. There were three time points for data collection—at baseline (when the caregiver agreed to participate), and at 1 month and 3 months after baseline.

### Measures and Outcomes

#### Primary Outcome

The primary outcome was related to an increase in the positive mental health score and a decrease in the caregiver burden score in the intervention group compared with the control group. This was measured using Lluch-Canut’s validated positive mental health questionnaire (PMHQ [42]). The PMHQ consists of 39 items distributed among 6 factors that describe positive mental health: F1–Personal satisfaction (8 items), F2–Prosocial attitude (5 items), F3–Self-control (5 items), F4–Autonomy (5 items), F5–Problem-solving and self-actualization (9 items), and F6–Interpersonal relationship skills (7 items). The items are in the form of positive or negative statements that patients rate on a scale from 1 to 4 according to how frequently they occur: 1=always or almost always, 2=quite often, 3=sometimes, and 4=rarely or never. The PMHQ provides a global score for positive mental health (sum of the item scores) as well as specific scores for each factor. The global positive mental health value ranges from 39 points (low positive mental health) to 156 points (high positive mental health). The minimum and maximum values for each factor are as follows: F1 8-32, F2 5-20, F3 5-20, F4 5-20, F5 9-36, and F6 7-28.

Caregiver burden was assessed using the 7-item short-form version of the Zarit Caregiver Burden Interview (ZBI-7), which was validated by Martín Carrasco et al [43] and Regueiro Martínez et al [44]. It measures the caregiver’s perceived burden in providing care. The 7 items are assessed on a 5-point Likert scale, ranging from 0=never to 4=almost always. Item scores are summed to obtain a total score ranging from 0 to 28, with higher scores indicating greater burden. The questions focus on major areas such as caregivers’ health, psychological well-being, finances, social life, and the caregiver-patient relationship.

#### Secondary Outcome

The secondary outcome was related to the usability and satisfaction regarding the app by the intervention group. An ad hoc questionnaire was administered by the nurse in charge. This questionnaire was created by the research group to evaluate the usability and satisfaction of the app-based intervention program. Qualitative data were also collected by the nurse through an open interview to obtain feedback on the user experience from the app-based intervention program users.

### Data Analysis

Descriptive statistics, including means for continuous variables and proportions for categorical variables, were used to summarize the characteristics of the participants. Analyses were conducted using an intention-to-treat analysis. Bivariate analysis was performed to calculate the mean change between the baseline and follow-up (at 1 month and 3 months). Next, to estimate the difference between the two groups, we calculated the difference between the mean change in the intervention group and the mean change in the control group. Due to the nonnormal distribution of the sample, nonparametric tests were used (Kolmogorov-Smirnov test, *P*<.001). To compare differences between the groups, the Mann-Whitney U test and Wilcoxon test were used. A *P* value ≤0.05 was considered significant. Cohen *d* analysis was performed to measure the effect size. Dropouts were not analyzed. Statistical analysis was performed using SPSS Statistics software (version 25 for Mac; IBM Corp).

If more than two items were missing from either of the instruments, the total score was not calculated and the data were considered missing. Missing data from the PMHQ and ZBI-7 were handled through average imputation of answered items, as long as no more than 40% of the items were missing. If more than 40% of the items were missing, the overall score was not calculated and the data were considered missing.

### Validity and Reliability/Rigor

CONSORT-EHEALTH (Consolidated Standards of Reporting Trials of Electronic and Mobile HEalth Applications and onLine TeleHealth) [45,46] was used to guide the design and implementation of the RCT. The researchers, who are experienced with clinical trials, monitored the study design, study protocols, patient recruitment, blinding, subject dropouts, and patient information confidentiality. The scrupulous study design ensured quality management and high external validity.

### Ethical Considerations

The study was approved by the Ethics Committee of Institut d’Atenció Primaria Jordi Gol (reference number: PI18/207). Caregivers were informed about the content, purpose, and procedure of the study. Written informed consent was given. Patients were reassured that their withdrawal would not prevent them from receiving the care that they would normally receive. The study was conducted in accordance with the principles of the Declaration of Helsinki, revised and updated, and followed Spain’s best practice guidelines (Buena Práctica Clínica). No negative impact was expected on the participants of the study. Data confidentiality was protected under the Spanish law governing the protection of personal data (Ley Orgánica 3/2018 de Protección de Datos de Carácter Personal). The participants were identified by research codes, and research information remained confidential.

## Results

### Recruitment in the RCT

A total of 152 caregivers were assessed for eligibility by the nurses in the primary health centers, and 131 agreed to participate and met the inclusion criteria. During the allocation into the intervention group, 4 caregivers were excluded because the app did not work properly on their smartphones. An additional 2 caregivers dropped out of the study because of the death of the person being cared for. Finally, 13 participants were lost to follow-up, including 7 participants in the intervention group who did not complete the app activities).

**Figure 3:**
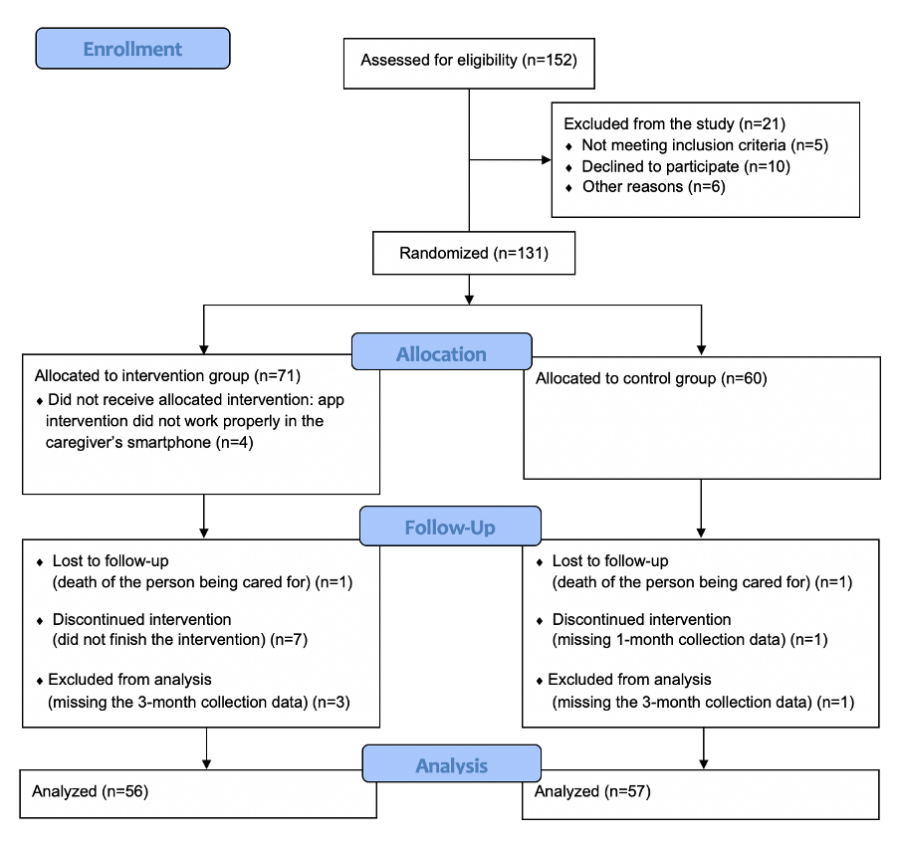
Participant flow in the study.

In all, 113 participants completed the trial: 56 in the intervention group and 57 in the control group. Thus, 79% (56/71) of the sample in the intervention group finished the intervention program. The loss to follow-up rate for the study was 13.7% (18/131) (Figure 3).

### Characteristics of the Participants

#### Caregivers

The baseline sociodemographic characteristics of the intervention and control groups are presented in Table 1. Overall, there was a large difference in the numbers of male and female caregivers in the study (8.0% versus 92.0%), which reflects the reality of the gender of caregivers. In terms of nationality, only 4.4% of caregivers in the sample were born outside of Spain. Marital status was similar between the groups. The relationship with the person being cared for and his/her situation of dependency was similar between the groups as well.

**Table 1:** Baseline sociodemographic characteristics of the intervention and control groups.

Sociodemographic characteristics		Control group (n=57)	Intervention group (n=56)	Total (N=113)
**Age (years)**				
	Mean (SD)	64.35 (13.49)	56.89 (13.49)	60.65 (12.37)
	Min-max	31-94	28-75	28-94
**Gender, n (%)**				
	Men	3 (5.3)	6 (10.7)	9 (8.0)
	Women	54 (94.7)	50 (89.3)	104 (92.0)
**Nationality, n (%)**				
	Spanish	55 (96.5)	53 (94.6)	108 (95.6)
	Other	2 (3.5)	3 (5.4)	5 (4.4)
**Level of studies, n (%)**				
	No studies	7 (12.3)	3 (1.8)	10 (7.1)
	Middle school	30 (52.6)	14 (25.0)	44 (38.9)
	High school	4 (7.0)	9 (16.1)	13 (11.5)
	Professional studies	5 (8.8)	12 (21.4)	17 (15.0)
	College	11 (19.3)	18 (32.1)	29 (25.7)
	No answer	0 (0.0)	2 (3.6)	2 (1.8)
**Marital status, n (%)**				
	Single	10 (17.5)	10 (17.9)	20 (17.7)
	Married	37 (64.9)	36 (64.3)	73 (64.6)
	Divorced	4 (7.0)	8 (14.3)	12 (10.6)
	Widowed	6 (10.5)	2 (3.6)	8 (7.1)
**Occupation, n (%)**				
	Unpaid job	14 (24.6)	6 (10.7)	20 (17.7)
	Paid job	22 (38.6)	28 (50.0)	50 (44.2)
	Retired	18 (31.6)	18 (32.1)	36 (31.9)
	Unemployed	3 (5.3)	4 (7.1)	7 (6.2)
**Do you have free time? , n (%)**				
	Yes	53 (93.0)	55 (98.2)	108 (95.6)
	No	4 (7.0)	1 (1.8)	5 (4.4)
**Type of caregiver, n (%)**				
	Primary	54 (94.7)	43 (76.8)	97 (85.8)
	Secondary	3 (5.3)	13 (23.2)	16 (14.2)
**Relationship with the person being cared for, n (%)**				
	Parent	33 (57.9)	39 (69.6)	72 (63.7)
	Partner	15 (26.3)	10 (17.9)	25 (22.1)
	Other but family-related (eg, grandparent, father/mother-in-law)	7 (12.3)	3 (5.4)	10 (8.8)
	Other with no family relation (eg, friend, neighbor)	0 (0.0)	2 (3.6)	2 (1.8)
	Nonprofessional caregiver contracted by the family or the person cared for	2 (3.5)	2 (3.6)	4 (3.5)

Some differences in variables between the groups had to be taken into consideration. Although age was taken into consideration during the randomization process to avoid an age bias, there was a considerable difference in the mean age of the groups (64.35 years versus 56.89 years in the control and intervention groups, respectively). This result was related to the 4 caregivers who did not receive their allocated intervention. Those 4 participants were over 65 years of age, and although they had smartphones, the phones did not have enough memory to support the app. Researchers considered that the 8-year age difference between the groups was reasonable considering the intended sample and its lower percentage among those who used smartphones in the control group. The participants’ level of studies also needs to be taken into consideration, as the intervention group had a greater percentage of individuals with a higher level of education. This difference could be related to the age difference. Regarding occupations, 50.0% (28/56) of participants in the intervention group had a paid job compared with 38.6% (22/57) in the control group. Another difference to be highlighted is that 23.2% (13/56) of participants in the intervention group were secondary caregivers compared with 5.3% (3/57) in the control group.

Caregivers were asked about their perceptions of their personal well-being and tasks. No major differences between the groups were found (Table 2).

**Table 2:** Characteristics of caregivers’ perceptions of their own well-being.

	Mean score (SD)^a^		
Characteristics	Control group (n=57)	Intervention group (n=56)	Total (N=113)
Perception of well-being	7.18 (1.48)	7.08 (1.48)	7.13 (1.39)
Level of burden	6.77 (2.08)	6.34 (2.08)	6.56 (2.32)
Level of satisfaction with caregiving tasks	7.61 (1.82)	7.98 (1.82)	7.80 (1.69)
Level of difficulty of caregiving duties	5.72 (2.66)	5.70 (2.66)	5.71 (2.50)
Level of the demands of the person being cared for	5.28 (3.18)	5.50 (3.18)	5.39 (3.06)

^a^Score from 0 to 10 (0=lowest score, 10=highest score).

#### Individuals Being Cared For

Caregivers were asked questions about the person in their care. The average age (control group: 84.88 years; intervention group: 81.63 years) and gender (control group: 61.4% women; intervention group: 67.9% women) of the individuals being cared for were similar between the groups (Table 3).

**Table 3:** Characteristics of individuals being cared for.

Characteristics		Control group (n=57)	Intervention group (n=56)	Total (N=113)
**Age (years)**				
	Mean (SD)	84.88 (8.95)	81.63 (10.16)	83.27 (9.66)
	Min-max	58-102	53-100	53-102
**Sex, n (%)**				
	Men	22 (38.6)	18 (32.1)	40 (35.4)
	Women	35 (61.4)	38 (67.9)	73 (64.6)
**Situation of dependence of the person being cared for, n (%)**				
	Multiple chronic conditions	25 (43.9)	35 (62.5)	60 (53.1)
	Alzheimer	14 (24.6)	10 (17.9)	24 (21.2)
	Fragility	12 (21.1)	4 (7.1)	16 (6.2)
	Stroke	5 (8.8)	2 (3.6)	7 (14.2)
	Tetraplegic	0 (0.0)	2 (3.6)	2 (1.8)
	Neoplasia	0 (0.0)	2 (3.6)	2 (1.8)
	Parkinson	0 (0.0)	1 (1.8)	1 (0.9)
	Schizophrenia	1 (1.8)	0 (0.0)	1 (0.9)

### Primary Outcome

The primary outcome was assessed using the PMHQ and ZBI-7. One of the main differences between the groups was related to the higher baseline PMHQ scores in the control group than in the intervention group. In fact, F2–Prosocial attitude, F3–Self-control, and F5–Problem solving and self-actualization are the factors behind this difference. The scores between the groups on the other factors were similar (Table 4).

**Table 4:** Descriptive results of the Positive Mental Health Questionnaire (PMHQ) (total scores and factor scores) and the 7-item short-form version of the Zarit Caregiver Burden Interview (ZBI-7).

Items		Control group (n=57)		Intervention group (n=56)	
		Mean (SD)	Range	Mean (SD)	Range
**PMHQ total score**					
	Baseline	120.10 (20.32)	84.00-152.00	98.60 (10.96)	51.00-127.00
	1 month	118.94 (20.05)	80.00-150.00	101.55 (14.70)	39.00-147.00
	3 months	121.68 (19.52)	85.00-156.00	114.41 (20.30)	85.00-152.00
**F1: Personal satisfaction**					
	Baseline	25.52 (4.38)	11.00-32.00	24.87 (4.47)	8.00-32.00
	1 month	24.60 (4.30)	11.00-31.00	25.59 (3.95)	9.00-30.00
	3 months	23.44 (5.37)	9.00-32.00	24.23 (6.17)	11.00-32.00
**F2: Prosocial attitude**					
	Baseline	16.07 (3.41)	9.00-20.00	11.21 (1.99)	6.00-20.00
	1 month	16.32 (3.72)	6.00-20.00	11.33 (2.51)	5.00-20.00
	3 months	16.84 (2.96)	10.00-20.00	14.98 (3.42)	9.00-20.00
**F3: Self-control**					
	Baseline	14.40 (3.81)	6.00-19.00	11.32 (2.35)	7.00-16.00
	1 month	14.75 (3.83)	7.00-20.00	11.24 (2.90)	5.00-20.00
	3 months	15.28 (3.51)	5.00-20.00	13.73 (3.25)	7.00-20.00
**F4: Autonomy**					
	Baseline	16.19 (3.07)	5.00-20.00	16.23 (3.08)	5.00-20.00
	1 month	15.54 (3.64)	5.00-20.00	16.40 (3.34)	5.00-20.00
	3 months	15.37 (4.21)	5.00-20.00	15.21 (4.52)	5.00-20.00
**F5: Problem solving and self-actualization**					
	Baseline	26.05 (8.49)	10.00-36.00	15.30 (5.26)	9.00-33.00
	1 month	26.46 (8.33)	9.00-36.00	15.11 (5.87)	9.00-36.00
	3 months	29.21 (6.77)	10.00-36.00	25.39 (8.76)	9.00-36.00
**F6: Interpersonal relationship skills**					
	Baseline	21.86 (3.43)	12.00-28.00	19.63 (2.93)	7.00-25.00
	1 month	21.29 (3.75)	12.00-28.00	19.75 (3.27)	7.00-25.00
	3 months	21.54 (4.29)	13.00-28.00	20.85 (4.12)	13.00-28.00
**ZBI-7^a^ score**					
	Baseline	19.77 (5.38)	9.00-29.00	18.80 (5.64)	7.00-32.00
	1 month	20.56 (5.24)	9.00-31.00	18.29 (5.34)	7.00-32.00
	3 months	20.70 (5.44)	8.00-32.00	17.69 (5.52)	7.00-29.00

^a^The ZBI-7 was completed by 92 caregivers (43 from the control group, 49 from the intervention group). The data were missing at random, with more than 40% of answer sheets being incomplete.

Assessments were conducted at baseline, and at 1 month and 3 months following the app intervention with the intervention group. Immediately after the intervention (Table 5), there were no statistically significant differences in changes in the PMHQ (*P*=.24) or ZBI-7 (*P*=.24) scores between the groups. There were statistically significant—but not clinically relevant—differences in the mean change in F1–Personal satisfaction (0.96; *P*=.03; *d*=–0.00) between the groups. Each group obtained statistically significant differences in ZBI-7 scores. While ZBI-7 scores decreased in the intervention group, they increased in the control group. However, there were no clinically relevant differences in the ZBI-7 scores between the groups.

**Table 5:** Comparison of mean changes at the 1-month follow-up between and within the intervention and control groups.^a^

Mean change at 1-month follow-up		Intervention group–control group		Intervention group		Control group	
		Mean difference of change (95% CI)	*P* value	Mean change (95% CI)	*P* value	Mean change (95% CI)	*P* value
**PMHQ^b^ total score**		1.40 (–3.98 to 5.72)	.24	0.37 (–4.09 to 4.83)	.21	–1.16 (–3.72 to 1.41)	.69
	F1–Personal satisfaction	0.96 (–0.62 to 2.47)	.03^a^	0.22 (–1.17 to 1.61)	.07	–0.93 (–1.89 to 0.32)	.20
	F2–Prosocial attitude	–0.07 (–1.08 to 0.68)	.18	0.11 (–0.46 to 0.68)	.92	0.25 (–0.24 to 0.74)	.17
	F3–Self-control	0.51 (–1.11 to 0.87)	.86	–0.1 (–0.89 to 0.85)	.98	0.35 (–0.43 to 1.13)	.31
	F4–Autonomy	0.79 (–0.38 to 1.79)	.14	0.22 (–0.82 to 1.27)	.17	–0.65 (–1.47 to 0.17)	.38
	F5–Problem solving and self-actualization	0.05 (–2.65 to 1.13)	.45	–0.26 (–1.45 to 0.93)	.35	0.40 (–0.93 to 1.73)	.95
	F6–Interpersonal relationship skills	0.77 (–0.71 to 1.86)	.18	0.09 (–0.99 to 1.18)	.51	–0.59 (–0.58 to 0.08)	.11
ZBI-7^c^ score		–0.55 (3.98 to 5.72)	.24	–0.51 (–1.51 to 0.49)	.05	0.79 (0.16 to 1.42)	.01

^a^Analyzed using Mann-Whitney U test and Wilcoxon signed rank test, with *P* values ≤.05 considered statistically significant.

^b^PMHQ: Positive Mental Health Questionnaire.

^c^ZBI-7: 7-item short-form version of the Zarit Caregiver Burden Interview.

The comparison of the mean changes at the 3-month follow-up (Table 6) revealed statistically significant differences in PMHQ scores between the intervention and control groups (11.43; *P*<.001; *d*=0.82). F5–Problem solving and self-actualization stood out for its clinically relevant mean difference of change (5.69; *P*<.001; *d*=0.71). However, F2–Prosocial attitude was the factor with the largest effect size (mean difference of change of 2.47; *P*<.001; *d*=1.18). F1–Personal satisfaction and F6–Interpersonal relationship skills had smaller effect sizes, but there were statistically significant differences between the groups (F1: 1.36; *P*=.05; *d*=0.36; and F6: 1.39; *P*=.04; *d*=0.25). F3–Self-control had a statistically significant difference and a moderate effect size (0.76; *P*=.03; *d*=0.50). F4–Autonomy did not show clinically relevant differences between the groups (–0.25; *P*=.99). ZBI-7 scores showed statistically significant differences with moderate clinically relevant results (–2.03; *P*<.001; *d*=–0.68).

**Table 6:** Comparison of mean changes at 3-month follow-up between and within the intervention and control groups.^a^

Mean change at 3-month follow-up			Intervention group–control group			Intervention group		Control group	
			Mean difference of change (95% CI)	*P* value	Cohen *d* (95% CI)^b^	Mean change (95% CI)	*P* value	Mean change (95% CI)	*P* value
**PMHQ^c^ total score**			11.43 (8.92 to 22.33)	<.001	0.82 (0.43 to 1.21)	14.94 (9.14 to 20.72)	<.001	3.51 (–1.54 to 8.56)	.78
	F1–Personal satisfaction		1.36 (–0.62 to 2.47)	.05	0.36 (–0.01 to 0.75)	–0.96 (–2.73 to 0.82)	.89	–2.32 (–4.41 to –0.24)	.01
	F2–Prosocial attitude		2.47 (–0.03 to 3.68)	<.001	1.18 (0.78 to 1.58)	3.73 (2.79 to 4.67)	<.001	1.26 (0.51 to 2.00)	.02
	F3–Self-control		0.76 (–1.61 to 1.87)	.03	0.50 (0.12 to 0.88)	2.31 (1.13 to 3.50)	<.001	155 (0.18 to 2.93)	.22
	F4–Autonomy		–0.25 (–0.78 to 1.79)	.99	–	–1.44 (2.78 to –0.10)	.26	–1.19 (–2.76 to 0.38)	.29
	F5–Problem solving and self-actualization		5.69 (–2.65 to 8.13)	<.001	0.71 (0.32 to 1.09)	10.29 (7.41 to 13.17)	<.001	4.60 (1.96 to 7.25)	.02
	F6–Interpersonal relationship skills		1.39 (–0.71 to 2.85)	.04	0.25 (–0.12 to 0.62)	1.00 (–0.30 to 2.30)	.03	–0.39 (–1.73 to 0.95)	.44
ZBI-7^d^ score			–2.03 (–5.07 to 1.36)	<.001	0.68 (–1.08 to –0.27)	–1.10 (–2.24 to 0.04)	<.001	0.93 (–0.08 to 1.93)	.04

^a^Analyzed using Mann-Whitney U test and Wilcoxon signed rank test, with *P* values ≤.05 considered statistically significant.

^b^Cohen *d* was only reported when the *P* value of the mean difference was statistically significant.

^c^PMHQ: Positive Mental Health Questionnaire.

^d^ZBI-7: 7-item short-form version of the Zarit Caregiver Burden Interview.

### Secondary Outcome

The level of satisfaction with the app was high. Questions 1 to 3 on the ad hoc questionnaire related to the operating system and had an average score of 93.9%. Regarding the organization of the app (questions 4 and 5), users did not report many difficulties in using the app. Users liked the daily phrases, claiming that they helped to improve their mental health (Q6: 43/49, 87.8%). Also, 100% (49/49) of users rated the activities as being easy (Q7), and 91.8% (45/49) of users found the TIVA character to encourage them to continue using the app (Q8). The majority of caregivers (46/49, 93.9%) responded that they would recommend the app and 27 of 49 (56.3%) users indicated that they felt the intervention should last longer (Table 7).

**Table 7:** Satisfaction with the app by the intervention group (n=49).^a^

Questions		Value, n (%)
**Q1: Was the installation of the app easy?**		
	Yes	41 (83.6)
	No	8 (16.4)
**Q2: Was the operation of the app well adapted to your mobile device?**		
	Yes	48 (98.0)
	No	1 (2.0)
**Q3: Was the response of the mobile app fast?**		
	Yes	49 (100)
	No	0 (0)
**Q4: Did the app provide information on the steps to follow?**		
	Always	42 (85.7)
	Frequently	4 (8.2)
	Sometimes	3 (6.1)
**Q5: Was there ever a time when you did not know what to do?**		
	Sometimes	13 (26.5)
	Never	36 (73.5)
**Q6: Do you feel that the daily phrases have helped to improve your mental well-being?**		
	Yes	43 (87.8)
	No	6 (12.2)
**Q7: Did you find the activities easy to do?**		
	Yes	49 (100)
	No	0 (0)
**Q8: Did the evolution of TIVA encourage you to continue using the mobile app?**		
	Yes	45 (91.8)
	No	4 (8.2)
**Q9: Would you recommend the app to other caregivers?**		
	Yes	46 (93.9)
	No	3 (5.4)
**Q10: Would you extend the intervention time?**		
	Yes	27 (56.3)
	No	21 (43.8)

^a^Of the 56 participants in the intervention group, app satisfaction data from 7 participants were lost.

## Discussion

### Principal Findings

This study aimed to asses the effectiveness of a digital intervention program to promote positive mental health among nonprofessional caregivers and to evaluate the usability and satisfaction of the app-based intervention program. The results demonstrated that the implementation of an app-based intervention program for caregivers significantly contributed to enhancing their positive mental health. In fact, results showed that the program produced a larger effect on the caregivers’ lives after 3 months of the intervention, which suggests that it is an effective long-term program. In addition, the participants in the study reported a high satisfaction rate with the app.

The intervention program seemed to be effective in relation to the differences in F1–Personal satisfaction scores between the groups at 1-month follow-up. Although the other factors did not reflect any significant differences between the groups, F4–Autonomy and F6–Interpersonal relationship skills obtained a positive mean difference of change, which reflects greater improvement of the scores in the intervention group. In addition to these program results, the level of burden of the control group seemed to increase after 1 month; in contrast, the ZBI-7 scores decreased in the intervention group, although the mean difference of change was not statistically significant.

The results seemed to be more satisfactory after 3 months of the intervention. There were significant differences between the groups on all factors, with the exception of F4–Autonomy, which was the only factor with scores that decreased from 1-month to 3-month follow-up.

The factor with the biggest increase after 3 months of intervention was F5–Problem solving and self-actualization. The mean change in the intervention group increased. These improvements could be related to the increase in confidence and caregiving information promoted by the program. A recent review about caregiver programs highlighted the importance of interventions that include program resolution strategies [47]. These strategies include the need for interventions to be easy and provide a connection with health care providers. Both elements are included in these app-based programs.

The app-based intervention program was also shown to be effective by comparing the scores of the intervention group on F2–Prosocial attitude, F3–Self-control, and F6–Interpersonal relationship skills with those of the control group. Programs involving group social support have already been tested and demonstrate their efficacy in caregivers. However, a few studies demonstrate that online strategies could promote those elements. In fact, a meta-analysis performed in 2017 highlighted the need to promote this type of program, as there are many isolated caregivers who cannot access on-site caregiver support programs [48]. The results of our study provide the first step in promoting this kind of program to foster prosocial attitudes through ICT strategies and with the support of primary health care nurses.

Another outcome of the study relates to F1–Personal satisfaction. In fact, this factor slightly increased in the intervention group after the first month, and decreased after 3 months. Therefore, interventions related to this factor are effective to prevent a further decrease over time. The research team believes that this result may be related to the messages of coaching and motivation that the app included, which were designed by the research team, including psychologists and nurses. Those motivational phrases were created with the aim of recognizing the caregiver’s task and its value.

In this section, it is important to highlight that after the app-based intervention program ended, users had the option of keeping the app on their smartphones, which would continue to display the question “Hello, how are you today?” every day. Furthermore, if they used the app, they could see the final evolution of TIVA and access the website [35]. Although not formally registered, this element seemed to satisfy the caregivers, who had made their request to the nurses responsible for data collection. A previous study shows that caregiver support is strongly related to their need for more care-related information and more information on available resources [49]. These needs could be related to our outcomes and to increased effectiveness in the intervention group after 3 months.

Results related to the ZBI-7 showed that after 3 months, the intervention group had a statistically significant decrease in caregiver burden, with a moderate size effect between the groups. However, researchers consider that these results are inconclusive considering the lack of information recorded in this study about the evolution of the person being cared for, which could have varied considerably after 3 months of intervention. Evidence shows how caregiver burden is directly associated with the caregiver’s duties, and further studies should evaluate the evolution of the characteristics of the caregiving situation [50,51].

The secondary outcome related to the app-based intervention program was satisfactory in terms of user acceptance. Users found the app easy to use, which was one of the goals of the app’s development. The literature highlights the need for these types of online programs to have an easy operating system to prevent dropouts [52,53]. According to informal feedback from caregivers and nurses, the character of the program, TIVA, was one of the reasons for the high degree of loyalty of the intervention program users to the app. In fact, many caregivers expressed several positive emotions after witnessing TIVA’s evolution every day.

Similar to applications designed for other types of users, new technologies designed to care for caregivers can increase satisfaction and motivation by empowering them, which promotes healthy changes in their behaviors and improves their quality of life [53]. Currently available applications have worked on aspects related more to the care of the chronic patient than to the well-being of the caregiver. With this application, and as recommended by other authors, behavioral interventions have been performed using “persuasive health technologies,” which are becoming much more promising approaches that encourage healthy behaviors [54]. Interventions that improve positive mental health should be an integral part of emotional support for caregivers and used in conjunction with advice from health professionals, improving their relationship without overloading face-to-face care. Most of these programs were conducted in a traditional face-to-face format. Nursing has the responsibility of initiating changes with the use of new technology and, as shown with the TIVA program, mHealth must be used as an indispensable working tool to reach all users, including those who cannot be reached in person [55].

Several studies emphasize the need to implement online intervention programs with gamification [21,52,53]. During the TIVA app’s creation, several options were considered to implement gamification in order to ensure the caregivers’ involvement during the 28 days of the intervention program. A game-based option was not considered, as it would have required more of the caregiver’s time and thus posed a risk of increasing dropouts.

On the other hand, the program allowed the self-management of time when planning the daily operation of the program. In this way, adherence was facilitated and the risk of dropouts was also reduced, as was also shown in another study [56].

Although the increase in the use of new technologies opens up a new space in the promotion of health for patients and caregivers, we identified in consonance with other authors that most of the existing digital support programs were more commercial than functional and it is necessary to promote studies that provide scientific evidence on the impact of these applications in the health field [57].

Therefore, the TIVA app seems to have been a good choice for this app-based intervention program. However, we can only express qualitative data on this outcome, so this should be assessed in further studies.

### Limitations and Future Work

A number of important issues remain to be addressed by future research. First, the basic characteristics of the participants showed some differences. There was an 8-year difference in the mean ages of the groups, which was related to individuals’ knowledge of how to use a smartphone. Further studies may need to compare the same age ranges with a minimum sample size for each group. This was not possible in our case, as the sample was not large enough to do this comparison. A similar problem occurred in relation to the level of education: the intervention group appeared to have a higher level of education than the control group. This could also have led to a bias in the results given that previous studies have linked the level of education to better coping strategies and level of resilience [58-60]. Despite these differences in sociodemographic characteristics, both groups obtained similar scores on characteristics of well-being and the person being cared for, which strengthens the validity of the results. Nevertheless, further studies with larger sample sizes should be undertaken to avoid possible biases.

Second, the level of positive mental health measured using the PMHQ appeared to be different at baseline for the intervention and control groups. The intervention group had a significantly lower level of positive mental health. This could have resulted in a bias in our study, as it might have been easier to show an improvement in the intervention group than in the control group. However, user satisfaction seems to be consistent in the results. Nevertheless, we believe that further studies need to be conducted to consider the baseline level of positive mental health, as well as the type of caregiver, as criteria to be included during the randomization process to verify the results.

Third, another limitation of the study is related to the type of caregiver. There was a higher percentage of secondary caregivers in the intervention group than in the control group. However, as the analysis was conducted with a consideration of pre- and postintervention scores, significant differences are valid.

Fourth, at the baseline of the study, caregiver burden was assessed using the ZBI-7. At the same time, data related to the characteristics of the person being cared for and information about the caregiving tasks (including the time per week dedicated to caregiving and caregiving duties) were recorded. However, while the ZBI-7 was also assessed after 1 month and after 3 months, the rest of the information was not. We believe that results related to caregiver burden should be carefully considered, and further studies should include an assessment of changes of caregivers’ duties and characteristics of the person cared for throughout the study.

Fifth, the app-based intervention program includes the features described in the “Methods,” which includes a total of 20 activities. Those activities, developed by the research team, were created with consideration of the 10 recommendations from Lluch-Canut’s model for promoting positive mental health [36,37]. Each activity cannot be linked directly to a factor from the model, as each activity promotes more than one factor. This is the reason why a redesign of the activities/features cannot be linked directly to a factor. Based on the results of this study, we consider, as a future line of research, a qualitative evaluation of the actual design of the app-based intervention program with the research team, stakeholders, and caregivers who participated in this study in the intervention group.

### Conclusions

The app-based intervention program analyzed in this study can be considered effective, with a high degree of user satisfaction. The development of a 28-day program with interventions based on Lluch-Canut’s positive mental health framework was successful in terms of user satisfaction and usability. The effectiveness of the program as it relates to positive mental health was demonstrated with positive results. However, we conclude that the program’s effectiveness should be verified by further studies where the baseline level of positive mental health and type of caregiver are included as randomization criteria.

This app-based intervention program showed increased positive mental health levels after 1 month and 3 months. The factors with the greatest long-term effect were F2–Prosocial attitude, F3–Self-control, and F5–Problem solving and self-actualization. However, activities related to F4–Autonomy should be revised, as the effectiveness was only maintained after 1 month and not after 3 months.

The app’s user satisfaction was encouraging. Users considered the app’s operational system and activities to be appropriate. The character created especially for the app, TIVA, with a programmed evolution after each activity, was considered to be a fundamental element to encourage users to continue with the app-based intervention program.

The results of the study encourage the promotion of app-based intervention programs for caregivers and endorse their effectiveness and user satisfaction. We believe that a further study involving stakeholders and participants should be considered to evaluate the adequacy of the activities in the actual app-based program in order to address the need to redesign activities/features of the app-based intervention program to increase the program’s effectiveness.

## Appendix

Multimedia Appendix 1 CONSORT-eHEALTH checklist (V1.6.1).

## Abbreviations

ICTs: information and communication technologies
mHealth: mobile health
PMHQ: Positive Mental Health Questionnaire
RCT: randomized controlled trial
ZBI-7: 7-item short-form version of the Zarit Caregiver Burden Interview
